# An International Proficiency Test to Detect, Identify and Quantify Ricin in Complex Matrices

**DOI:** 10.3390/toxins7124859

**Published:** 2015-11-26

**Authors:** Sylvia Worbs, Martin Skiba, Jennifer Bender, Reinhard Zeleny, Heinz Schimmel, Werner Luginbühl, Brigitte G. Dorner

**Affiliations:** 1 Biological Toxins, Centre for Biological Threats and Special Pathogens, Robert Koch Institute, Seestrasse 10, 13353 Berlin, Germany; worbss@rki.de (S.W.); skibam@rki.de (M.S.); benderj@rki.de (J.B.); 2 Joint Research Centre, Institute for Reference Materials and Measurements, European Commission, Retieseweg 111, 2440 Geel, Belgium; reinhard.zeleny@ec.europa.eu (R.Z.); heinz.schimmel@ec.europa.eu (H.S.); 3 ChemStat, Aarstrasse 98, 3005 Bern, Switzerland; info@chemstat.ch (W.L.)

**Keywords:** proficiency test, ricin, reference material, standardized detection

## Abstract

While natural intoxications with seeds of *Ricinus communis* (*R. communis*) have long been known, the toxic protein ricin contained in the seeds is of major concern since it attracts attention of those intending criminal, terroristic and military misuse. In order to harmonize detection capabilities in expert laboratories, an international proficiency test was organized that aimed at identifying good analytical practices (qualitative measurements) and determining a consensus concentration on a highly pure ricin reference material (quantitative measurements). Sample materials included highly pure ricin as well as the related *R. communis* agglutinin (RCA120) spiked into buffer, milk and meat extract; additionally, an organic fertilizer naturally contaminated with *R. communis* shred was investigated in the proficiency test. The qualitative results showed that either a suitable combination of immunological, mass spectrometry (MS)-based and functional approaches or sophisticated MS-based approaches alone successfully allowed the detection and identification of ricin in all samples. In terms of quantification, it was possible to determine a consensus concentration of the highly pure ricin reference material. The results provide a basis for further steps in quality assurance and improve biopreparedness in expert laboratories worldwide.

## 1. Introduction

The plant toxin ricin produced by *Ricinus communis* (*R. communis*) has been intensively studied since its identification in 1888 by Stillmark [1]. Ricin is a prototype AB toxin of approximately 60 kDa consisting of a catalytically active A-chain (~32 kDa) which acts as an RNA *N*-glycosidase and a sugar-binding B-chain (lectin, ~34 kDa) linked via a disulfide bond [2,3]. Cell binding occurs through the B-chain and involves different oligosaccharide residues on the cell surface. Several oligosaccharide residues, including *N*-acetylglucosamine and galactose residues on glycolipids and glycoproteins, are known receptors for the lectin subunit, and these oligosaccharides show a broad and abundant presence on mammalian cells [4,5,6]. The study of ricin (RCA60) was complicated by the presence of a homologous protein in the seeds of *R. communis* identified as *Ricinus communis* agglutinin (RCA120), a much less toxic dimeric protein with high sequence identity to ricin. Whereas ricin is a monomeric AB toxin, *R. communis* agglutinin is a ~120 kDa dimer of two A- (~32 kDa) and B-subunits (~36 kDa) [7] in which the two A-chains are linked by a disulfide bond [8]. The amino acid sequences of the A- and the B-chains of RCA60 and RCA120 show a high degree of homology of 94% and 85%, respectively [9]. This reflects their similar but not identical structures and biochemical properties [10,11]. Adding further complexity to the issue, an isoform of ricin named ricin E (while the original ricin is now termed ricin D) was later discovered both on protein and on DNA levels to contain a hybrid B-chain of ricin and *R. communis* agglutinin [12,13,14].

Recently, sequence analysis methods have revealed that ricin and *R. communis* agglutinin are members of a ricin gene family encoding seven full-length ricin or ricin-like proteins and several potential shorter gene products of unknown expression and function, reflecting a much greater variability as previously anticipated [15,16]. The full-length proteins of the ricin gene family have been shown to inhibit protein synthesis similar to ricin itself [16]. Additional heterogeneity of ricin is based on different glycosylation patterns [17] and variable toxicities of ricin isoforms have been correlated with different glycosylation levels [18,19]. Another level of complexity has recently been added by the description of heterogeneity in the deamidation pattern, the conversion rate of single asparagine residues to aspartic and isoaspartic acid [20].

Ricin and the ricin-producing plant are recognized as dual-use substances: On the positive side, *R. communis* is of economic interest for the production of castor oil and the numerous industrial, medical and cosmetic products derived from it [3,21]. Castor oil contains high levels of the unusual fatty acid, ricinoleic acid, which is rewarded for its unique chemical properties used in the production of lubricants, pharmaceuticals, paints, coatings, inks and other products. Furthermore, the ability of the A-subunit to induce cell death has been exploited for the development of immunotoxins and medical application [3,22,23]. The catalytic A-chain of ricin was one of the first examples of a toxin coupled to monoclonal antibodies against cell surface proteins and tested experimentally for the treatment of various cancers [3,24,25,26,27]. On the negative side, ricin has a history of military, criminal, and terroristic misuse. It was included in different weapons programmes during World War II under the codename “compound W”, and weaponized material was later produced until the 1980s [3,28,29,30,31]. Therefore, ricin is a prohibited substance both under the Chemical Weapons Convention (CWC, schedule 1 compound) and the Biological Weapons Convention (BWC) and its possession or purification is strictly regulated and controlled by the Organization for the Prohibition of Chemical Weapons (OPCW) [3]. The relative ease in preparing a crude extract and the world-wide availability of the plant has also made ricin a potential agent of bioterrorism, therefore it is listed as category B agent of potential bioterrorism risk by the Centers for Disease Control and Prevention [3,32,33]. In the past, the focus fell on the toxin for criminal misuse and various attempted acts of bioterrorism; for example, the “ricin threat letters” sent in 2003 and 2013 to members of the US Senate and the White House as well as to U.S. President Obama gained broad media coverage [28,34].

Due to the toxin’s potential for misuse, the rapid, sensitive and ideally unambiguous detection of ricin is necessary. A range of different detection methods are available using immunological, spectrometric, functional or molecular approaches [3]. Antibody-based immunoassays such as enzyme-linked immunosorbent assays (ELISAs) belong to the most sensitive routine technologies with detection limits between a few ng/mL and fg/mL depending on the antibodies used [35,36,37,38,39,40,41]. Since classical ELISAs require several hours for analysis fast on-site detection systems such as hand-held lateral flow assays (LFA) [41,42,43] and automated biosensor technologies have been developed [44,45,46] which usually provide results within 30 min down to a few ng/mL of toxin. Currently, immunological assays are not able to distinguish ricin from the related RCA120, a task that might be relevant in the course of a forensic investigation. In this context, modern mass spectrometry methods (e.g., Matrix-Assisted Laser Desorption Ionization—Time of Flight mass spectrometry (MALDI-TOF MS) and liquid chromatography-electrospray ionization-tandem mass spectrometry (LC-ESI MS)) are able to deliver unambiguous sequence information from pure and crude toxin preparations, and sensitivities can reach down to a few ng/mL of toxin when a combination of immunoaffinity- or lectin-based enrichment, tryptic digestion plus MS-based detection and identification of specific peptides is applied [47,48,49,50,51,52,53]. Additionally, different functional approaches have been introduced which detect the A-chain activity (e.g., adenine-release assays, cell free translation assays [54,55,56,57,58,59,60,61], the B-chain activity (e.g., enzyme-linked lectin assays [62]) or both (e.g., cell-based cytotoxicity assays [60,63,64,65,66]). Current cell-based assays use different endpoint read-outs of cell death via biochemical, fluorescent or radioactive detection [63,64,65,66] or, alternatively, display the cytotoxic activity of ricin in real-time based on impedance measurement [60]. The detection limits for ricin analysis in cell-based bioassays have been described as being between 0.01 ng/mL and 0.8 ng/mL from complex matrices. Alternatives include assays where an antibody-based enrichment is combined with adenine release measurement by mass spectrometry [51,67,68].

While different technologies for ricin detection and identification have been established, no universally agreed “gold standards” are available. Expert laboratories currently use differently purified in-house reference materials for quantification, making any comparison of accuracy and sensitivity of different methods nearly impossible. The aim of the proficiency test (PT) on ricin organized in the framework of the EU-project EQuATox (Establishment of quality assurance for the detection of biological toxins of potential bioterrorism risk, www.equatox.eu; funded under the European Community’s Seventh Framework Programme) was to provide an overview and evaluation of existing methods for screening and identification of ricin. Herein we describe selected qualitative and quantitative PT results obtained by 17 international expert laboratories from 12 countries. The results highlight “best practices” for the analysis of ricin and are an important step towards harmonization and standardization of analytical methods.

## 2. Results and Discussion

### 2.1. Preparation of the Ricin Proficiency Test

To set up a proper PT test plan, nine samples were selected for further preparatory analysis taking into account the following (Table 1):

(i) The samples needed to be detectable with a range of different techniques, as the PT was open with respect to the methods applied by the participants. The expectation of a technically open PT was to obtain information on best analytical practices. To this end, three different concentrations of highly purified ricin (prepared and characterized in [69]) in buffer containing a stabilizing protein were selected: a high (500,000 ng/mL), an intermediate (500 ng/mL) and a low (0.5 ng/mL) concentration of purified ricin in PBS/0.1% BSA.
(ii) For the analyses of the influence of complex matrices on the detection of ricin, the intermediate concentration of ricin (500 ng/mL) was spiked into semi-skimmed milk and a particle-free, sterile extract of minced meat.
(iii) To obtain information on the specificity of different methods, the highly homologous RCA120 was selected (prepared and characterized in [69]). Equivalent concentrations of RCA120 and ricin (500,000 ng/mL and 500 ng/mL) were spiked into buffered solutions (PBS/0.1% BSA).
(iv) Finally, as “real sample”, a commercially available organic fertilizer containing *R. communis* shred was used that caused a case of dog poisoning in Germany [3]. This material represented a naturally contaminated sample containing unknown concentrations of ricin, RCA120 and the alkaloid ricinine. According to the manufacturer the fertilizer was enriched with a crude *R. communis* preparation, the press-cake of an industrial castor oil extraction process that is often used as additive in fertilizer as a rich source of nitrogen. The sample was included in the PT to evaluate the laboratories’ sample preparation strategies.

The PT samples, as depicted in Table 1, were further analyzed by stability testing. According to Thompson *et al.* and ISO/IEC 17043:2012, samples have to be sufficiently stable during the predefined testing period which was set to four weeks by the PT organizer [70,71]. Stability testing was performed by two sandwich ELISAs detecting ricin or RCA120 [69], using ten aliquots of each of the nine samples depicted in Table 1. Five aliquots were stored at −80 °C for four weeks, and for comparison five aliquots were stored at 4 °C for four weeks, the latter representing the recommended storage condition during the PT. All sample sets were analyzed simultaneously by either ricin and/or RCA120-ELISAs corresponding to the toxin contained in the respective sample.

**Table 1:** PT test plan.

Samples Selected as Potential PT Samples for Further Stability Testing		PT Sample Number
1	Negative sample (=buffer: 0.1% BSA/PBS)	S1
2	500,000 ng/mL of ricin in 0.1% BSA/PBS	S6
3	500 ng/mL of ricin in 0.1% BSA/PBS	S3
4	0.5 ng/mL of ricin in 0.1% BSA/PBS	S7
5	500 ng/mL of ricin in semi-skimmed UHT milk	S4
6	500 ng/mL of ricin in extract of minced meat	S8
7	500,000 ng/mL of RCA120 in 0.1% BSA/PBS	S2
8	500 ng/mL of RCA120 in 0.1% BSA/PBS	S5
9	Organic fertilizer (solid sample material)	S9

**Figure 1:**
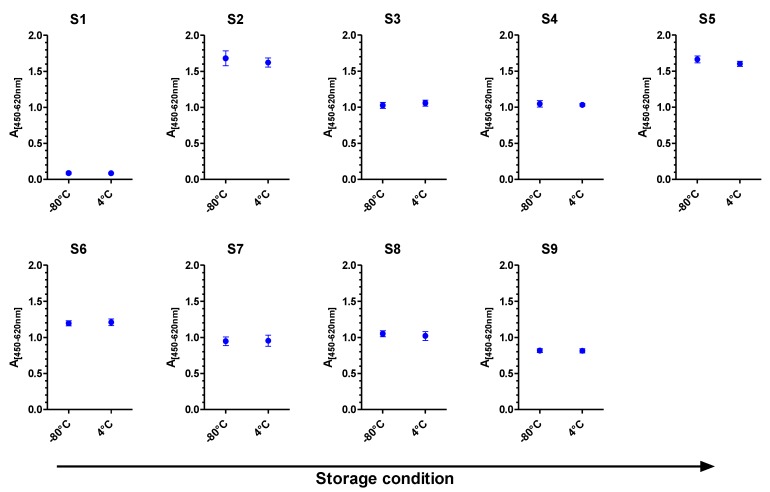
Stability of PT samples as measured by sandwich ELISA. Five replicates of each of the nine samples were either stored at −80 °C or 4 °C for four weeks. Absorbance of samples S1, S3, S4, S6, S7, S8 and S9 was measured by ricin-ELISA, absorbance of samples S2 and S5 by RCA120-ELISA. Plotted is the absorbance at 450 nm minus absorbance at the reference wavelength 620 nm against the storage condition −80 °C or 4 °C for four weeks; error bars indicate the standard errors obtained for five randomly selected sample replicates per storage condition.

As shown in Figure 1, the results indicated that all samples were sufficiently stable over the given time period of four weeks. This result was confirmed statistically by Dunnett’s tests which showed no significant deviation in concentrations under these two storage conditions (all *p* > 0.05; not shown).

**Figure 2:**
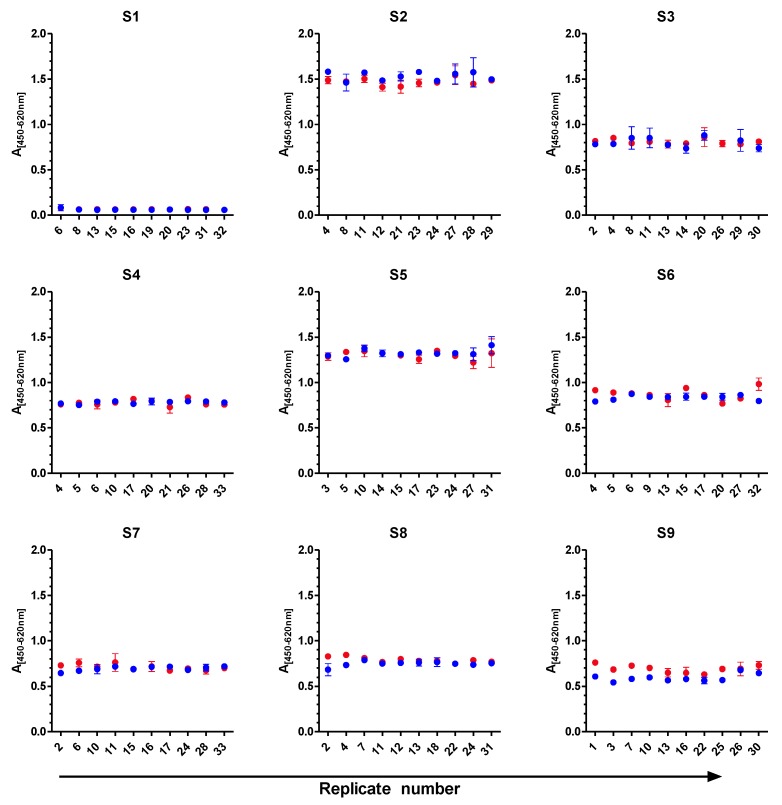
Homogeneity study. Ten randomly selected test portions of each sample (S1–S9) were analyzed by a sandwich ELISA preferentially detecting ricin or RCA120, respectively, in two independent experiments (depicted in red and blue), each performed in duplicate. The mean absorption of each duplicate with its standard error (error bars) is plotted against the ten replicates of each sample. Absorbance of samples S1, S3, S4, S6, S7, S8 and S9 were measured by ricin-ELISA, absorbance of samples S2 and S5 by RCA120-ELISA.

Based on the stability study, samples S1 to S9 were selected as suitable PT samples. For the actual PT, 33 aliquots of each sample S1 to S9 were prepared as described before. Of these, ten aliquots of each sample were randomly selected for homogeneity testing. Homogeneity of each test material was assessed according to Thompson *et al.* [71] and ISO/IEC 17043:2012 [70] by employing the corresponding sandwich ELISAs for either ricin or RCA120. Figure 2 graphically displays the results of the homogeneity tests in which ten aliquots of each sample were measured twice in duplicate by ELISA. At first glance measured absorbance values indicate sufficient sample homogeneity. It was noticeable that in some cases the standard deviation of duplicates was larger than the variation between the two experiments (e.g., S3). Statistically, Cochran tests showed outlying variances in four samples (one only in each of the four samples) at a significance level of 0.05 (not shown). In accordance with Recommendation 9 in [71] these variance outliers were excluded from the assessment of the homogeneity according to Recommendations 7 and 8 in [71]. This assessment proved sufficient homogeneity for each sample.

Although the highly purified ricin and RCA120 preparations used to spike the PT samples and thoroughly characterized by Worbs *et al.* [69] represent well-defined, qualified materials, they are still not certified as reference materials. According to Thompson *et al.*, in this situation it is necessary to determine the protein concentration experimentally after spiking the purified toxins into the buffer or matrix [71]. This provides the “nominal concentration” of the samples as opposed to the “theoretical concentration” that is the known spiked concentration, assuming there are no losses during sample preparation, no matrix effects or other disturbing factors.

Therefore, the ricin- and RCA120-ELISA were used as above to precisely quantify all nine samples: three randomly selected aliquots of each sample were measured in independent experiments in duplicate on three consecutive days. The mean absorption values of the duplicates that lay in the linear range of the standard curve were interpolated in the standard curve to calculate the concentrations of the samples. The calculated concentrations of the three replicates of each sample measured on three days were statistically analyzed, and estimates of the nominal concentrations were obtained with the robust algorithm according to ISO 5725-5:1998 [72] as depicted in Table 2.

**Table 2:** Proficiency test: sample identity and statistics. Nominal concentrations are highlighted in bold. Consensus mean concentrations based on the participants’ reported results used as *x_a_* are highlighted in green.

Sample	Matrix	Measurand	c(Theoretical) *	c(Nominal) **	σ(rob)	x_a_	σ_p_	Unit
S1	0.1% BSA/PBS	-	-	-	-	-	-	-
S2	0.1% BSA/PBS	RCA120	500,000	**572,851**	62,686	**563,994**	143,876	ng/mL
S3	0.1% BSA/PBS	Ricin	500	**504**	110	**522**	133	ng/mL
S4	skimmed milk	Ricin	500	**473**	96.3	**436**	111	ng/mL
S5	0.1% BSA/PBS	RCA120	500	**445**	65.2	**481**	123	ng/mL
S6	0.1% BSA/PBS	Ricin	500,000	**589,508**	78,055	**588,949**	150,242	ng/mL
S7	0.1% BSA/PBS	Ricin	0.5	**0.414**	0.112	**0.441**	0.112	ng/mL
S8	meat extract	Ricin	500	**484**	111	**508**	130	ng/mL
S9	Organic fertilizer	RCA120	-	**42**	5.818	**42**	52.6	µg/g
		Ricin	-	**306**	71.6	**206**	10.7	µg/g

* The “theoretical concentration” was the known concentration of ricin or RCA120 that was spiked into the different matrices. Sample S9 was a naturally contaminated material, the true “theoretical values” were not known. ** Robust estimates of mean nominal concentrations as determined experimentally by the organizing laboratory by ELISA for ricin or RCA120, respectively. σ(rob): robust estimate of the standard deviation of the nominal concentrations. *x_a_*: assigned value σ_p_: standard deviation for proficiency assessment.

Additionally, for the subsequent quantitative analysis of PT results reported by the participants, the assigned values *x_a_* for the nine samples were defined according to the following decision rule: the consensus mean based on the participants’ reported results was used as *x_a_* if the absolute difference between the nominal value determined in the organizer’s laboratory and the mean of the participants’ responses was not larger than 50% of the nominal value given in Table 2 (this was the case for all but one quantitative measurement); otherwise the nominal value was used. Based on *x_a_* the standard deviation for proficiency assessment, σ_p_, was calculated assuming a normal variate 0.95 confidence interval of (*x_a_* − 0.5·*x_a_*; *x_a_* + 0.5·*x_a_*) (corresponding to a reproducibility limit of 0.5·*x_a_*), *i.e.*, σ_p_ = 0.5·*x_a_*/1.96 = *x_a_*/3.92 (Table 2). As there are no “true” values or certified reference materials available, this was the choice made on the basis of the rule that inter-laboratory reproducibility limits are very often about twice the repeatability limits. The latter was assumed to be about 25% of the concentration, as was supported later by the experience in this PT. Table 2 summarizes the theoretical concentration for each sample, the robust estimate of the mean nominal concentration based on the experiments performed in the organizer’s laboratory, the robust estimate of the standard deviation of the nominal concentrations as well as the assigned values and the standard deviation for proficiency assessment for each sample and measurand.

For samples S1–S8 the theoretical concentration was known, and the experimentally determined nominal concentrations ended up closely to the expected theoretical concentration. The only exception was sample S9, the organic fertilizer, a real sample material, where no theoretical values for ricin and RCA120 concentration were known.

Determination of the nominal concentrations of the nine samples concluded the preparatory experimental part of the ricin PT. With respect to shipment of active toxin-containing samples and depending on the destination of the shipment, an OPCW notification as well as different individual authorizations were required and obtained by the national authorities of the participating countries, e.g., clearance certificates and import or export permits. The actual shipment was realized using a dedicated shipper (World Courier, Germany) as security transport: the transport of toxins as a dangerous goods shipment followed the classification toxic class 6.1, UN3172. The material was packed in IATA/ADR-approved 4GU boxes (Bio-Bottles, Alex Breuer GmbH, Cologne, Germany), and the dispatch of samples from the organizer’s laboratory was done by a certified shipping agent. The samples were transported in Bio-Bottles securely locked in 20 kg steel containers equipped with temperature loggers and cooling devices and were tracked throughout the shipment.

Two months before the actual shipment of samples, the interested laboratories obtained an official announcement letter including a nomination form and information on objectives of the PT, the test design, the potential sample materials and measurands, a timeline for the PT, basic information on reporting and analysis as well as comments on the requirements and regulations to be obeyed. Deadline to deliver results was announced to be four weeks after shipment of samples. 17 expert laboratories from 12 countries worldwide actively took part in the exercise and received 1.2 mL of liquid samples S1 to S8 and 10 g of solid sample S9. All samples reached their destination within three days. The electronically transmitted temperature logging files indicated that all packages arrived at their destinations at temperatures below 7 °C. The participants confirmed that all samples arrived cooled and in a good condition.

In order to re-confirm sample stability, the organizer’s laboratory measured once again the concentration in all samples in one randomly selected sample set four weeks after sample shipment (not shown). The concentrations determined in the *post* stability test were compared to the nominal concentrations determined before sending samples to PT participants in the homogeneity study and confirmed the findings of the *pre* PT stability studies, *i.e.*, that all samples were sufficiently stable during the period of the ricin PT.

### 2.2. Results of the Ricin Proficiency Test

One major goal of the ricin PT was to define good analytical strategies; therefore, the PT was open with respect to the methods applied by the PT participants. The participants were asked to deliver their results both qualitatively and/or quantitatively in two technically independent replicates (including all steps of sample preparation) per method applied, using a dedicated Excel reporting file. Additionally, since laboratories were free to combine different methods and analytical approaches, they were asked to fill in a report summarizing their sample-specific conclusions in a final result sheet, taking into account different results that might have been obtained by applying different methods. One challenge in this international PT laid in the restricted sample volume provided (1.2 mL for S1–S8/10 g for S9). If a laboratory was planning to apply both qualitative and quantitative analysis or to combine different technical approaches the volume per analysis had to be carefully planned. Qualitative and quantitative results reported by the PT participants were summarized in anonymized form and selected results will be discussed in the next two sections.

#### 2.2.1. Qualitative Results of the Ricin Proficiency Test

Participants were asked to report their experimental results as “ricin”, “RCA120”, “ricin and/or RCA120”, “negative result (*i.e.*, nothing detected)” or “not analyzed” in a dedicated Excel workbook. Qualitative results were assessed according to the degree of trueness of the participant’s assignments and color codes were used to indicate the assessment (Table 3). Samples S2 to S8 were assessed as “correct/light green” if results were reported as “ricin and/or RCA120” without differentiation between ricin and RCA120 taking into account the following consideration: in case of an intentional release of toxins from *Ricinus communis* it would be important to detect the material as fast and reliable as possible in order to take adequate actions. Depending on the scenario, ricin and RCA120 would potentially be found together in the sample material. In a potential biothreat scenario, it is important to know which methods would be able to detect the threat and to identify “dangerous” samples, irrespective of the differentiation of the two highly homologous proteins. The differentiation of ricin and RCA120 is technically challenging, but might be necessary under certain circumstances (e.g., in the context of OPCW activities or in a case of prosecution). Therefore, if PT participants were able to differentiate ricin from RCA120 for samples S2 to S8, this result was assigned as “completely correct/dark green”.

**Table 3:** Color code to represent correct assignment of the PT samples.

	Completely correct; for samples S2–S8 differentiation of ricin and RCA120
	Correct; no differentiation of ricin and RCA120
	Partly correct; one of two replicates was correct but not both
	Insufficient assignment
	Not analyzed

Generally, a variety of methods was applied in the ricin PT, combining different principles of detection, identification and quantification. Qualitative results reported by the participants on samples S1–S9 were analyzed by comparison of different technological approaches applied. Since a number of methods combine different analytical principles (e.g., immunoaffinity enrichment plus mass spectrometric detection and/or functional testing of ricin’s depurination activity) it was not easy to form well-defined groups; however, some general conclusions on the methods applied could be drawn by subdividing into immunological, mass spectrometric and functional methods.

**Figure 3:**
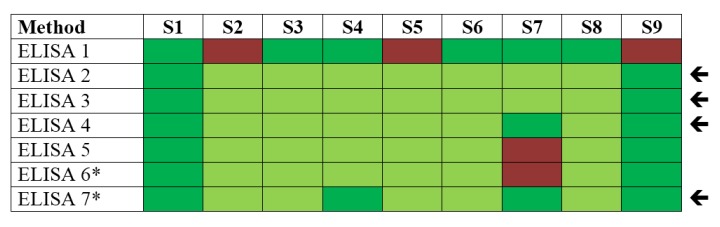
Qualitative results reported as “ricin”, “RCA120” and “ricin and/or RCA120” for all nine samples displayed by different ELISA protocols used. Sample S1 was the negative control sample, samples S3, S4, S6–S8 contained ricin, samples S2 and S5 contained RCA120, and S9 was the organic fertilizer containing *Ricinus communis* (both ricin and RCA120). Methods marked by an arrow delivered qualitatively correct results on all samples analyzed. Qualitative results reported by the participants were color-coded as indicated in Table 3; * results have been taken from the laboratory’s quantitative reporting since they accidentally have not been reported qualitatively.

**Figure 4:**
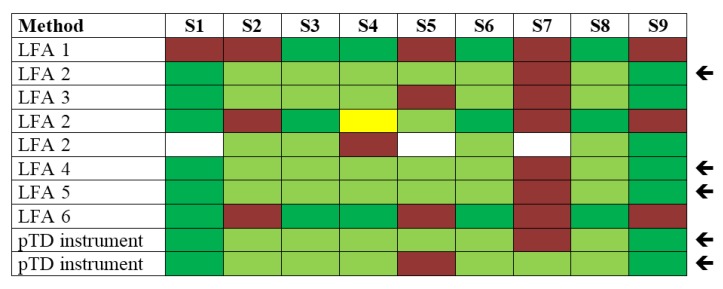
Qualitative results reported as “ricin”, “RCA120” and “ricin and/or RCA120” for all nine samples displayed by different on-site detection methods. Sample S1 was the negative control sample, samples S3, S4, S6–S8 contained ricin, samples S2 and S5 contained RCA120, and S9 was the organic fertilizer containing *Ricinus communis* (both ricin and RCA120). Methods marked by an arrow delivered qualitatively correct results on eight out of nine samples analyzed. Qualitative results reported by the participants were color-coded as indicated in Table 3.

With respect to immunological methods applied within this PT, seven different plate-bound sandwich ELISA formats based on different antibodies and detection protocols were used, among them in-house assays and commercial products (Figure 3). Additionally, two immunological on-site detection methods were applied: (i) six different LFA tests, including commercial products; and (ii) a commercially available electrical biochip sensor technology (portable Toxin Detector, pTD, Bruker Daltonik, Billerica, MA, USA; [44]) was used by two laboratories (Figure 4).

Figure 3 shows that most ELISAs delivered correct results on all nine samples. This was especially true for ELISA 2, 3, 4 and 7 (marked by an arrow). Flaws occurred on single samples using ELISA 1, 5 and 6. Notably, the different ELISAs were generally not able to discriminate ricin from RCA120, corresponding to the light green color code displayed in Figure 3 for samples S2–S8. On the positive side, the majority of ELISA approaches used were able to correctly identify sample S7, the sample with lowest concentration (0.441 ng/mL ricin), as ricin-containing material.

The results obtained for the LFAs were more heterogeneous than the ELISA results shown above (Figure 4). Three laboratories were able to detect eight out of nine samples correctly using LFA 2, LFA 4 and LFA 5 (marked by an arrow), the only flaw occurred for the sample S7 containing the lowest concentration, which obviously contained ricin below the detection limits of the assays (usually in the low ng/mL-range [43,73,74]). Generally, high and intermediate concentrations of ricin in buffer or complex matrices (S6, S3; S4, S8) were correctly detected by LFA. Some participants misinterpreted the RCA120-containing samples S2 and S5 as ricin. One commercially available product, LFA 2, was applied in three participating laboratories. Remarkably, this simple on-site detection test delivered three different results, possibly due to application or reporting errors in the three laboratories. It has been observed before that LFAs which are advertised as “easy to use assays” require a basic level of training and evaluation before reliable results can be obtained [73]. Furthermore, a previous study reported a significant variability in assay results with different commercial products: in a direct comparison only three out of six commercial LFAs tested performed well and delivered useful results [73]. LFA 5, the “BioThreat Alert Test Strip” distributed by Tetracore, USA, used in this exercise by one laboratory, has been identified before as robust, well-performing assay in independent laboratories [73,75]. LFA 2 and LFA 4, which performed well upon correct handling in this exercise, are presented in more detail together with four classical ELISA approaches (Figure 3) in Simon *et al.* in this Special Issue of Toxins [42].

In an alternative approach, two participants used the commercial pTD platform, an automated electrical biochip instrument based on miniaturized, multiplexed sandwich ELISAs performed on gold electrodes [44]. Similar to the LFA results, eight out of nine samples were correctly detected by this instrument (marked by an arrow). As with the classical ELISA (Figure 3), both the different LFAs and the pTD instrument were not able to discriminate ricin from RCA120 (corresponding to color code light green for S2–S8), which is of minor importance in an acute threat scenario. Nevertheless, this exercise identified the pTD instrument as well as LFA 2, LFA 4 and LFA 5 as suitable screening approaches to detect “dangerous”, ricin-containing samples. Further method-performance studies focusing on selected on-side detection assays applied in this exercise will have to be performed to obtain a more detailed insight into the general reliability and applicability of the approaches.

**Figure 5:**
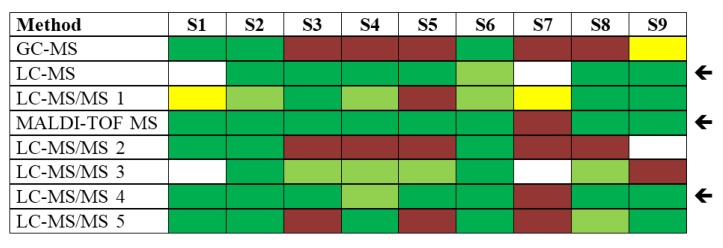
Qualitative results reported as “ricin”, “RCA120” and “ricin and/or RCA120” for all nine samples displayed by different MS-based approaches. Sample S1 was the negative control sample, samples S3, S4, S6–S8 contained ricin, samples S2 and S5 contained RCA120, and S9 was the organic fertilizer containing *Ricinus communis* (both ricin and RCA120). Methods marked by an arrow delivered qualitatively correct results on seven or eight out of nine samples analyzed. Qualitative results reported by the participants were color-coded as indicated in Table 3.

With respect to mass spectrometry-based approaches, different techniques and instrumentations were used (MALDI-TOF MS, LC-ESI MS). Most laboratories applied a combination of immunoaffinity enrichment using different antibodies with tryptic digestion and MS or MS/MS detection and identification (Figure 5). Three approaches delivered correct results on seven or eight out of nine samples analyzed. These are marked by an arrow in Figure 5.

In contrast to the immunological methods, these successful approaches delivered completely correct results straightaway—corresponding to color code dark green for S2 to S8—for the majority of the samples analyzed. Thus, in contrast to immunological assays selected spectrometric approaches allowed for unambiguous identification of the measurand. However, due to the detection limit of current MS approaches [51], the detection of sample S7 containing the lowest concentration of ricin was unsuccessful in this exercise. Selected successful mass spectrometric methods for the detection and identification of ricin are described in more detail by Kalb *et al.* in this Special Issue of Toxins [76].

With respect to functional testing, two different types of functional assays were applied by the laboratories:

(i) a combination of immunoaffinity enrichment plus detection of the depurination activity of ricin or RCA120 from an artificial substrate (MS-based adenine release assay);
(ii) a cell-based cytotoxicity assay detecting the cell death induced by ricin or RCA120.

**Figure 6:**
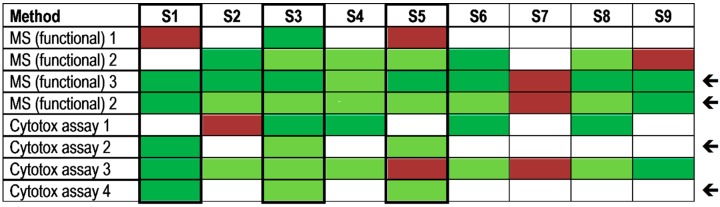
Qualitative results reported as “ricin”, “RCA120” and “ricin and/or RCA120” for all nine samples displayed by different functional approaches. Laboratories used either functional MS-based approaches to measure the ricin-induced adenine release by an artificial substrate or different cell culture-based cytotoxicity assays. Sample S1 was the negative control sample, samples S3, S4, S6–S8 contained ricin, samples S2 and S5 contained RCA120, and S9 was the organic fertilizer containing *Ricinus communis* (both ricin and RCA120). Methods marked by an arrow delivered qualitatively correct results on the samples S1, S3 and S5 which were specifically asked to be ranked by functional activity (optional task). Qualitative results reported by the participants were color-coded as indicated in Table 3.

As an optional challenge, the PT participants were asked to rank samples S1, S3 and S5 by their functional activity using the designation “highest”, “intermediate” and “lowest” functional activity. Therefore, functional assays were preferentially used on samples S1, S3 and S5 in order to work on the optional task, but a number of laboratories performed functional testing on more samples. Figure 6 shows qualitative results of the functional testing for all samples with respect to detection of sample content. Four approaches delivered correct results on samples S1, S3 and S5, among them two adenine release assays (MS, functional) and two cytotoxicity assays (marked by an arrow in Figure 6). The successful functional MS-based approaches detected eight out of nine samples correctly as ricin-containing material. Sample S7 containing the lowest ricin concentration was not detected by any of the functional tests applied.

**Figure 7:**
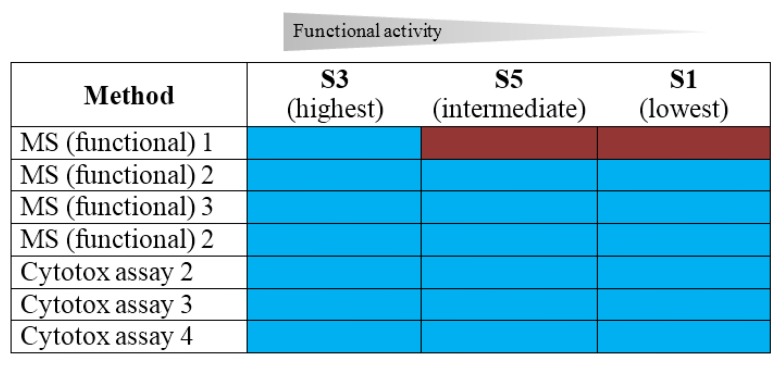
Results reported for the ranking of specified samples S1, S3 and S5 according to their functional activity by different methods. Laboratories used either functional MS-based approaches to measure the ricin-induced adenine release by an artificial substrate or different cell culture-based cytotoxicity assays. Sample S3 contained ricin and showed the highest functional activity; S5 contained RCA120 and had intermediate activity; S1 was the negative control sample without detectable functional activity. Results of ranking according functional activity reported by the participants were color-coded with blue “sample activity ranked correctly” and red “wrong ranking of sample activity”.

With respect to ranking samples S1, S3 and S5 by their functional activity into “highest”, “intermediate” and “lowest” functional activity, samples S3 and S5 contained ricin or RCA120, respectively, in intermediate concentrations (S3: 522 ng/mL ricin; S5: 481 ng/mL RCA120), while sample S1 was the negative control. Though the protein concentration of S3 and S5 was approximately the same, the difference in depurination activity and overall cytotoxicity could be correctly assigned: in the exercise, five out of six methods applied delivered correct results with respect to functional activity ranking (Figure 7). Successful protocols for functional testing are described in more detail in [60,67,68,76].

Based on the qualitative PT results reported by the 17 participating laboratories, good analytical practices can now be derived. To achieve this, the different technical approaches applied were grouped together according to their detection principle and statistically analyzed, to provide an overview of success rates obtained for the different detection principles on all nine samples. This analysis is useful for drawing some general conclusions on the methods applied, while it is important to keep in mind that different analytical protocols and tools have been used and that for each detection principle successful individual strategies have been identified in the PT (Figure 3, Figure 4, Figure 5 and Figure 6). Additionally, one should keep in mind that the methods were applied by a variable number of laboratories, so that statistics should not be overestimated. Table 4 provides an overview of success rates obtained for the different detection principles on all nine samples. Generally, the success rate of the methods applied ranged from 71.5%–77.7% when all nine samples were considered corresponding to similar overall success rates for the different methods (Table 4). At first sight, this outcome is different to results obtained in a parallel PT on botulinum toxin detection that was performed in the framework of EQuATox and is reviewed in Worbs *et al.* [77]: for this toxin, a set of methods was identified that resulted in higher success rates than other methods.

However, a more detailed analysis of ricin PT results considering individual samples allowed us to draw further conclusions (Table 5). To this end, mean success rates for different technical approaches were calculated for individual samples containing ricin or RCA120 in equivalent concentrations (e.g., comparison of samples S6 and S2 containing high concentrations of ricin or RCA120, respectively, and samples S3 and S5 containing intermediate concentrations of ricin or RCA120, respectively; Table 5). In this approach, the different immunological methods (e.g., ELISA, pTD and LFA; Figure 3 and Figure 4) were grouped together; similarly, the MS-based methods targeting sample identity—but not functional activity—were grouped together (e.g., MALDI-TOF MS and LC-MS/MS; Figure 5); finally, functional approaches were grouped together (e.g., cytotoxicity assay, functional MS assay; Figure 6; similar classification as in Table 4). As shown in Table 5, this comparative analysis helped to visualize advantages and limitations of different technical approaches. Comparing samples containing high concentrations of either ricin (S6) or RCA120 (S2), mass spectrometric approaches delivered superior results with success rates of 79%–87% indicating that these methods are best suited for differentiation of the highly homologous proteins. A mixed picture was obtained at intermediate concentrations with no clear trends for different technical approaches: ricin detection was achieved at similar success rates (50%–63%) using immunological, MS-based and functional approaches, with a tendency that MS-based and functional methods are somewhat better suited for the correct assignment of RCA120. At low ricin concentrations, however, immunological methods clearly delivered the highest success rates (21% compared to success rates of 0%–8% for functional and MS-based approaches); this is most probably linked to their higher sensitivity especially in conventional ELISA-formats (Figure 3; [38,42,43,51,74,78]).

**Table 4:** Qualitative results of methods used for ricin detection: overview of success rates obtained for different methods on all samples *. Numbers in bold highlight the total percentage of correct results (= sum of correct positive and correct negative results).

Main Assay Principle	Method	Total Number of Results	Number of Laboratories	Correct Results	%	Total %
**Immunological Method**	**ELISA**	103	7	correct positive	64.1	**77.7**
				correct negative	13.6	
	**On-site detection** (LFA, pTD)	165	10	correct positive	60.6	**71.5**
				correct negative	10.9	
**MS-Based Method**	**MS detection**	117	8	correct positive	42.7	**73.5**
				correct negative	30.8	
**Functional Method**	**Functional MS assay** (Adenine release)	29	4	correct positive	62.1	**75.9**
				correct negative	13.8	
	**Cytotoxicity assay**	35	4	correct positive	51.4	**74.3**
				correct negative	22.9	

* Detailed classification of success rates for the different methods applied for ricin detection. For each method the total number of results reported per method is indicated, the number of laboratories applying an individual method and the percentage of correct positive or correct negative results.

**Table 5:** Mean success rates given in percent obtained for different technical approaches on selected samples for analytes “ricin” or “RCA120” *.

Main Assay Principle #	S6 Ricin_high_ (588,949 ng/mL)	S2 RCA120_high_ (563,994 ng/mL)	S3 Ricin_intermediate_ (522 ng/mL)	S5 RCA120_intermediate_ (481 ng/mL)	S7 Ricin_low_ (0.441 ng/mL)
**Immunol. method**	71	43	63	27	21
**MS-based method**	87	79	50	39	8
**Functional method**	43	57	54	42	0

* Analyte concentration and sample number as indicated in the Table header. # Grouping of methods: ELISA, LFA and pTD results were grouped into “immunological method”, MALDI-TOF MS and LC-MS/MS results into “MS-based method” and cytotoxicity assay and functional MS assay into “functional method”.

This analysis might be helpful to decide which methods can be used and combined in order to get preliminary, confirmed, and unambiguous results on a ricin-containing sample. In this context, it is of particular note that several laboratories delivered correct results on all nine samples by combining different analytical approaches (*i.e.*, immunological, MS-based and functional methods) while individual laboratories were successful by combining different highly sophisticated MS approaches targeting both protein identity and functional activity of ricin. With respect to good analytical practices this information will be crucial in the future to develop recommended operating procedures and optimized workflows for the analysis of toxin-containing samples that are supported internationally.

#### 2.2.2. Quantitative Results of the Ricin Proficiency Test

Independent of the qualitative reporting, the participating laboratories were asked to perform quantification of ricin in the nine samples and to report the results of two independent measurements in a dedicated Excel reporting file. Again, for quantification any method established and validated in the laboratories was admitted; if the laboratories planned to use different methods for quantification, they were asked to submit results in separate quantitative reporting sheets. In this context, some basic questions were asked regarding the scope of assay validation performed prior to the PT (e.g., detection limit of the method, coefficients of variation and measurement uncertainty) and the reference material used. The quantitative measurements reported by the participants were—as far as possible—evaluated statistically according to the recommendations by Thompson *et al.* and Algorithm A of the international standard ISO 13528:2005 “Statistical methods for use in proficiency testing by inter-laboratory comparisons” [71,79].

Quantification of ricin in the PT samples was performed by 14 of 17 participating laboratories (some reported results as measurand “ricin”, others as measurands “ricin and/or RCA120”; only results reported as “ricin” are considered here). Most laboratories used classical ELISA-based methods for quantification, one laboratory used an MS-based approach (please see [42,76] for details). In order to assess and visualize quantitative results, *z*-scores were calculated according to the equation.

(1) $$z=\frac{x-x_{a}}{\sigma_{p}}$$

with *x* denoting the results reported by the participants, *x_a_* the assigned concentration value, and σ_p_ the standard deviation for proficiency assessment, respectively (Table 2). *z*-scores (in the context of PTs) quantify the difference between an individual single or mean result and the assigned value in units of the standard deviation for proficiency assessment. This transformation is known as standardization; the standardized dataset has a mean of zero (0) and a standard deviation (and variance) of one (1) if *x_a_* and σ_p_ are the respective statistics of the empirical distribution of the data considered. A *z*-score of zero indicates an unbiased result with respect to the assigned value, a *z*-score of 1 is one standard deviation for proficiency assessment above the assigned value, a *z*-score of −1 is one standard deviation for proficiency assessment below the assigned value and so on. Provided that the data points are realizations of normally distributed random variables with mean *x_a_* and standard deviation σ_p_ (*i.e.*, *x* ~ *N*(*x_a_*, σ_p_^2^)), this is the model to which the results reported are compared, the *z*-scores represent realizations of random variables of the standard normal distribution (*i.e.*, *z* ~ *N*(0, 1)) where about 95% of *z*-scores will fall between −2 and +2 (the sign “−” or “+” of the score indicates a negative or positive deviation, respectively). According to Thompson *et al.* [71], scores in this range are commonly designated “acceptable” or “satisfactory”. Scores in the ranges −2 to −3 and +2 to +3 would be expected about once in 20, and scores in this class are sometimes designated “questionable”. A score outside the range from −3 to +3 would be very unusual and is taken to indicate that the cause of the event should be investigated and remedied, and Thompson *et al.* [71] suggest the phrase “requiring action” for such results.

**Figure 8:**
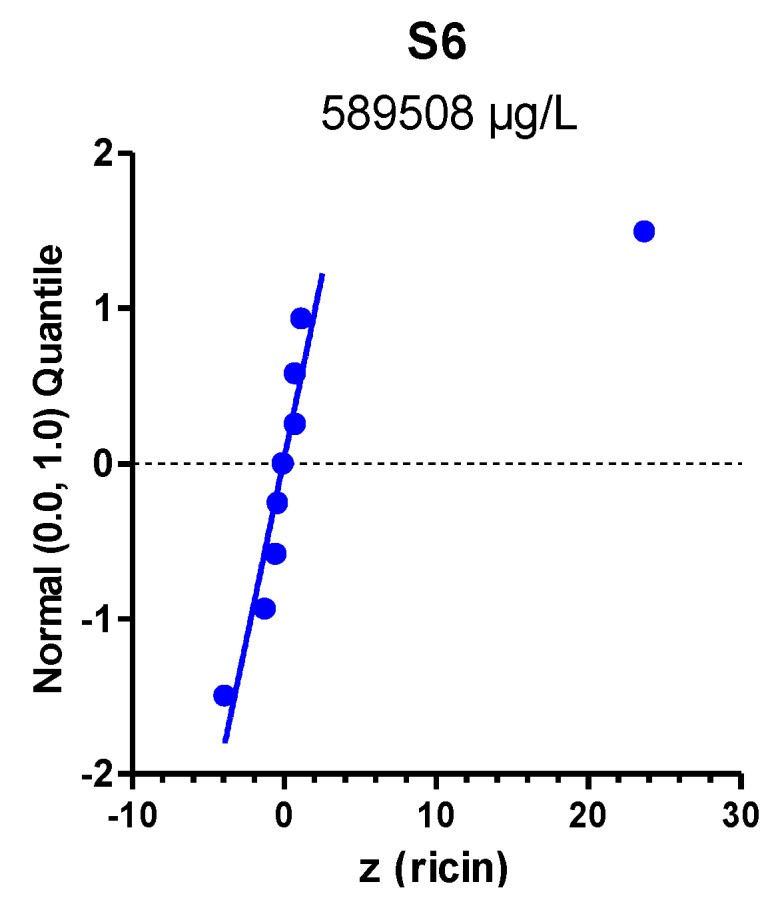
Normal probability plot of *z*-scores for quantification of ricin in sample S6. Standard normal quantiles were plotted against the *z*-scores to visualize if scores (representing concentrations reported) were normally distributed. The analysis was done by considering all methods used to quantify the indicated sample. Each dot corresponds to one method used by one laboratory; some laboratories used more than one method for quantification.

Exemplarily, the quantitative results provided by the participants for sample S6 containing the highest concentration of ricin in buffer is visualized in Figure 8 as normal probability plot of *z*-scores.

*z*-scores of normally distributed concentration values with sample statistics *x_a_* and σ_p_^2^ would lie along a straight line with slope 1 in these plots. Normal probability plots are used to display both the dispersion of a dataset and the deviation of the empirical distribution from statistical normality. As can be seen in Figure 8, most of the quantitative data of sample S6 approximately followed the normal distribution of the model *x* ~ *N*(*x_a_*, σ_p_^2^), only a single value was far off. Notably, all but one *z*-score obtained by the participants on sample S6 were close to zero. This was independent of the methods used for quantification—ELISA or MS-based method—and the individual reference material used in the laboratories. Seven quantitative results reported lay within the interval −2 < *z* < +2 corresponding to satisfactory results (Figure 8). Therefore, the mean of participants’ quantitative results for S6 as estimated by robust statistics was defined as consensus concentration of the ricin reference material generated in [69] and used to spike PT samples in this exercise.

**Figure 9:**
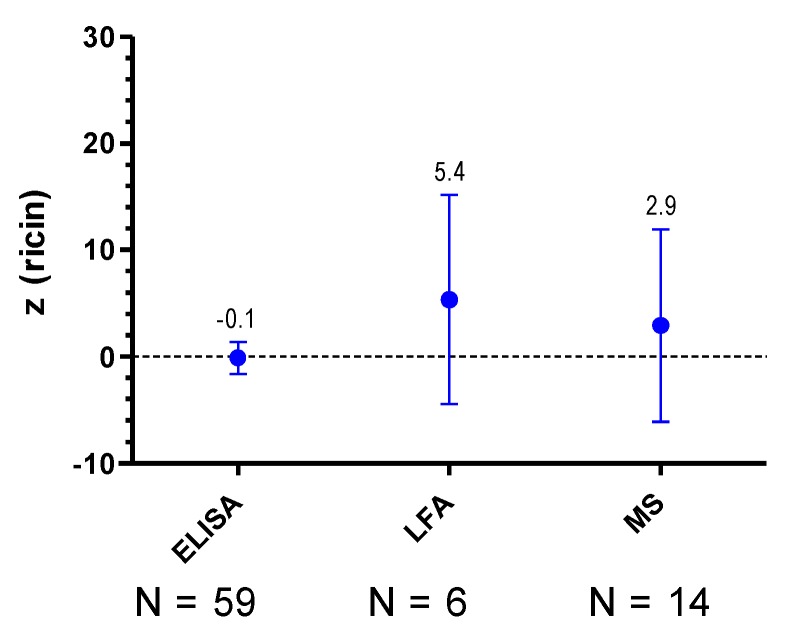
Accordance of methods. *z*-score means (points and figures) and their standard deviations (error bars span mean ± standard deviation) are depicted as computed from the *z*-scores specifically for indicated methods (*X*-axis) for the quantification of ricin. *N* indicates the number of *z*-scores available for each method analyzed in the given category. Analysis was done by considering all samples analyzed in the PT with the indicated methods.

In order to evaluate the methods used for quantification of ricin the accordance of methods was assessed based on the quantitative results reported and the calculated *z*-scores. Figure 9 shows the *z*-score means (points and figures) and their standard deviations (error bars span mean ± SD) as computed from the *z*-scores. The *z*-score means offer a guide to assess the mean closeness of a method to the assigned concentration if applied by a number of laboratories to a number of samples, and the corresponding standard deviation measures the variation of the *z*-scores among the respective samples and laboratories. *N* indicates the number of *z*-scores available for each method analyzed (please note that the number of available *z*-scores is variable). The analysis summarized in Figure 9 was performed by considering quantitative results reported for all samples in the PT.

The various classical ELISA formats used in this PT showed a good mean trueness (trueness in the sense of comparability of methods) with a *z*-score mean of −0.1 for all samples (*n* = 59, Figure 9) and a *z*-score mean of +0.1 when only sample S6 was considered (*n* = 12; not shown); furthermore, a good agreement of *z*-scores among the samples and laboratories corresponding to a small standard deviation was observed. Taking into account that different ELISA-formats based on different antibodies and different ricin reference materials were used in the laboratories, the quantitative results obtained by ELISAs indicate the superiority of this methodology.

For LFA and MS-based methods, less *z*-scores were available for this analysis, so caution has to be taken to draw general conclusions. Available data could be interpreted in the way that other methods used for quantification than ELISA showed larger deviations from the assigned values (*z*-scores > 2.5) and less accordance between the *z*-scores among samples and laboratories (high standard deviation; Figure 9). However, this effect might well be related to the reference material used and might be independent of the methods itself. This question should be further evaluated in future exercises.

## 3. Experimental Section

### 3.1. Preparation of PT Samples

As complex food matrices ultra-high temperature (UHT) semi-skimmed milk and minced meat were purchased from a local retail store. Milk was opened under sterile conditions and spiked with a defined amount of ricin or RCA120 as indicated in Table 1. A particle-free meat extract was prepared by extracting 10 g of minced meat from pork and beef (1:1) with 90 mL of gelatin phosphate buffer (pH 6). The meat was removed by centrifugation and the supernatant was autoclaved, filtrated and spiked with a defined amount of toxin as indicated above. In parallel, buffer (0.1% BSA/PBS pH 7) was spiked with toxin as indicated in Table 1. All spiked samples were analyzed and quantitated for their ricin or RCA120 concentration by sandwich ELISA without any further sample preparation (Section 3.2). For sample S9 an organic fertilizer naturally contaminated with *R. communis* shred was used that was involved in a case of dog poisoning in Germany [3]: to extract and quantify toxins contained in the fertilizer 2 g of the sample were extracted with 20 mL PBS (pH 7) for 2 h by rotating at room temperature. Supernatant was collected by centrifugation and quantification of ricin and RCA120 in this material was performed as described in Section 3.2.

### 3.2. Amplified Sandwich ELISAs for Ricin and RCA120

The ELISA specific for ricin was performed as described before in Pauly *et al.* [38,42]. Briefly, MaxiSorp microtiter plates (Thermo Fisher Scientific, Rockford, USA) were coated with 5 µg/mL of capture mAb R109 in 50 µL PBS overnight at 4 °C. Blocking was performed with casein buffer (Senova, Jena, Germany) for 1 h at room temperature. After a washing step, 50 µL of toxin-containing solution was added: (i) either serial dilutions of ricin reference material (an in-house purified ricin preparation, independently prepared of the material described in [69]) starting from 100 ng/mL to 0.05 pg/mL in assay buffer (PBS, 0.1% BSA (Sigma-Aldrich, Munich, Germany)) as standard curve; or (ii) diluted samples (dilution in PBS + 0.1% BSA). Samples were incubated for 2 h at room temperature. Detection was performed by incubation with biotin-labelled secondary antibody diluted in casein buffer (1 h, room temperature), followed by a washing step and detection with Streptavidin-PolyHRP40 conjugate (0.5 ng/mL, Senova, Jena, Germany). After a washing step the sandwich ELISA was developed by adding 100 µL of substrate solution 3,3ʹ,5,5ʹ-tetramethylbenzidine (TMB; SeramunBlau slow, Seramun Diagnostika, Heidesee, Germany). Reaction was stopped by 100 µL of 0.25 M acid sulfur.

The ELISA for RCA120 was performed similarly using mAb ARK4 as capture antibody (kindly provided by Marc-André Avondet, Spiez Laboratory, Switzerland; [80]) and biotinylated polyclonal chicken IgY RC22 [81] as detection antibody. As reference material for quantification the highly purified RCA120 described in [69] was used. Further information on the performance of both ELISA is given in [69].

### 3.3. Stability and Homogeneity Testing, Nominal Concentration

In order to demonstrate sample stability during the PT test period (set to four weeks), ten aliquots of each sample S1 to S9 were prepared by spiking of the matrices with ricin or RCA120 as indicated in Table 1 and used prior to the actual PT for stability testing. Five aliquots were stored for four weeks at −80 °C, and five aliquots were stored for four weeks at 4 °C for comparison (for a total number of 90 aliquots). After storage at the indicated condition the samples were frozen at −80 °C until analysis. All sample sets were analyzed simultaneously on a single day by the ELISA corresponding to the measurand contained in a sample: (i) an ELISA detecting ricin for samples S3, S4 and S6–S9; and (ii) an ELISA detecting RCA120 for samples S2, S5 and S9 (Section 3.2).

For analysis, all ricin-containing samples were diluted to a concentration of 0.1 ng/mL which is in the linear range of the respective ELISA close to the EC_50_ value. Along the same line, for the RCA120-ELISA samples containing RCA120 were diluted to a concentration of 0.5 ng/mL before analysis.

For statistical analysis of the ELISA results, two outlying values were identified by Grubbs tests (R and R package “outliers”) and excluded from the fitting of linear models with storage conditions as fixed effects and *post hoc* Dunnett tests with storage condition 4 w/−80 °C as control group, using SYSTAT 13 [82,83,84].

For homogeneity testing, 33 aliquots of each sample S1 to S9 were prepared as before, and ten randomly selected aliquots were used for homogeneity testing. Homogeneity of each test material was assessed according to Thompson *et al.* [71] and ISO/IEC 17043:2012 [70] on the basis of absorbance values at 450 nm obtained by sandwich ELISA. The ten randomly selected test aliquots of each sample were analyzed in duplicate in two independent experiments using the ELISA detecting ricin or RCA120, respectively. The ELISAs were performed as described in Section 3.2. Statistically, Cochran tests were performed to assess the homogeneity of variances; duplicates with outlying variances were excluded. As the data structure was more complex than described in Thompson *et al.* and therefore not suitable for the standard statistical procedure recommended in [71], factorial linear mixed models were set up to fit the data and to provide estimates of the sampling standard deviations and the analytical standard deviations, respectively, using SYSTAT 13 [83]. These variance components were assessed according to Thompson *et al.* [71], Recommendations 7 and 8.

In order to determine the nominal concentrations of the samples three aliquots of each sample S1–S9 were measured in duplicate in independent experiments on three days. The ELISAs were performed as described in Section 3.2. Extreme values were not excluded for the analysis; instead, the analytical results were evaluated by the robust algorithm according to ISO 5725-5:1998 and ISO 13528:2005 Annex C (using R package “metrology”) to compute the nominal concentrations [72,79,85]. For the determination of assigned values and the decision rule used please see Section 2.1 and Table 2.

### 3.4. Statistical Analysis and Data Visualization

Qualitative responses (categories “1”–“5”, indicating 1 = ricin, 2 = RCA120, 3 = ricin AND/OR RCA120, 4 = negative result and 5 = not analyzed) were compared to the correct answers by simple algorithms. For dichotomic grouping (correct/false) it was sufficient to introduce auxiliary variables using codes 1 and 0, respectively; codes for more differentiated evaluations were either multi-valued or built from the dichotomic codes. On the basis of these categorical auxiliary variables the success rates were obtained by frequency tabulation or by computing the means of the auxiliary variables grouped by the categories of interest (e.g., grouped by method). The resulting data tables were exported from SYSTAT to Excel^®^ and color-coded by conditional color formatting of each cell of the auxiliary variables.

The quantitative measurements reported by the participants were statistically evaluated according to the recommendations of Thompson *et al.* and Algorithm A of the international standard ISO 13528:2005 [79]. Statistical evaluations were performed using SYSTAT 13 and R (libraries “metRology” and “outliers”) [82,83,85].

Robust algorithms of the R package “metrology” were used to compute the assigned concentrations. *z*-scores were obtained on the basis of these assigned values (Table 2) and the respective standard deviations for proficiency assessment (see Section 2.1). Normal probability plots of the *z*-scores were produced by commercial software (SYSTAT 13; Figure 8: GraphPad Prism 5) in order to visualize the empirical distributions of the results reported, as compared to the model implicitly set as normal distribution with mean *x_a_* and variance σ_p_^2^ (*i.e.*, *x* ~ *N*(*x_a_*, σ_p_^2^)). Assessment of the accordance of methods was based on the arithmetic means of the individual *z*-scores as shown in Figure 9.

## 4. Conclusions

Ricin is recognized as a dual-use substance: On the one hand the ricin-producing plant is of economic interest for the production of castor oil and the numerous products produced using the oil. On the other hand, the ricin toxin, a byproduct of castor oil production, has a known history of military, criminal and terroristic misuse [21,28,29,30,31,32,33,34]. In addition, accidental intoxications in humans and animals have been reported [3]. The toxin is a list 1-compound under the CWC, therefore handling of ricin requires special attention and is strictly controlled by national and international authorities. The security and health concerns require the rapid detection, precise identification and accurate quantification of ricin in order to enable appropriate management decisions. Currently expert laboratories use different technologies for the detection and identification of ricin based on immunological, spectrometric or functional approaches, but hardly any universally agreed “gold standards” are available, including common internationally recognized reference materials, widely accessible tools, or accepted standard operating procedures. Differently purified in-house materials of variable quality are used in expert laboratories for validation purposes, making a direct comparison of accuracy, sensitivity, and specificity of different methods nearly impossible. Additionally, no regular training and self-evaluation possibilities such as PTs or ring trials on dedicated methods have been available in the past. In this respect the situation is clearly different from other scientific areas, e.g., the food sector, where regular demonstration of technical performance by proficiency testing or ring trials is generally accepted to accompany appropriate standardization efforts and to ensure technical competence. As examples, several proficiency tests and/or ring trials were performed in the food sector to test detection capabilities for low molecular weight toxins such as aflatoxins, mycotoxins and paralytic shellfish poisoning toxins, as well as high molecular weight toxins such as staphylococcal enterotoxins [86,87,88,89,90].

Against this background, the aim of the ricin PT conducted in the framework of the EQuATox project was to provide an overview and evaluation of existing methods for screening, detection, identification, and quantification of ricin among 17 participating laboratories from EU-28 and beyond. Nine samples were selected to test for sensitivity (different concentrations of ricin in buffer), specificity (differentiation of corresponding concentrations of ricin and the highly homologous RCA120) as well as matrix interference (spiked food matrices and a naturally contaminated organic fertilizer material [3]). A variety of methods were used by the participants, combining different detection principles. Qualitative results reported by the participants were analyzed by the different methods applied and the degree of trueness of the participants’ assignments.

Immunological methods applied included seven classical ELISA formats, six LFA formats and the pTD platform [44], the latter two technologies were tested as rapid on-site detection approaches. Four out of seven classical sandwich ELISA delivered correct results on all nine samples. Notably, these ELISA were able to detect sample S7 containing the lowest ricin concentration, which did cause difficulties when analyzed by other technical approaches. Detection of ricin in the low pg/mL range is relevant in the context of human ricin intoxications (own unpublished data and [91]). While the ELISA-based methods were not suitable for unambiguous identification of ricin, they were most sensitive to detect ricin-containing samples in the low pg/mL range which is usually not covered by other approaches. The pTD instrument as well as three LFA turned out to be suitable screening approaches to detect “dangerous”, ricin-containing samples within 20 min, albeit at reduced sensitivity compared to laboratory-based ELISA (eight out of nine samples identified correctly), the latter point is in accordance with literature data [35,36,37,38,39,40,41,42,43,73,75,92]. More detailed information on selected immunological methods is given in this Special Issue of Toxins by Simon *et al.* [42].

Common to all immunological methods was the inability to differentiate ricin from the highly homologous RCA120, a task that might be relevant under certain circumstances (e.g., an OPCW investigation or a lawsuit). In this context mass spectrometric methods to identify the protein content proved their ability to unambiguously discriminate between ricin and RCA120: three different MS-based methods were identified that gave correct results on seven or eight out of nine samples. Due to their currently limited sensitivity compared to ELISA-based methods, the low-concentration sample (S7) was not correctly identified [38,42,43,51,74,78]. However, MS-based methods offer the technical advantage of providing extensive detail about known and unknown sample contents, especially when using tandem mass spectrometry, thereby adding an open view into the diagnostic or forensic workflow [33]. Successful MS-based approaches are further described in [76] in this Special Issue of Toxins.

With respect to functional methods, two general approaches were used in the ricin PT: either a combination of immunoaffinity enrichment plus detection of the depurination activity of ricin from an artificial substrate (MS-based adenine release assay), or a cell-based cytotoxicity assay detecting the cell death induced by ricin. Two protocols each allowed to identify functionally active ricin or RCA120 and to rank three specified samples according to their functional activity. Successful protocols for functional testing are described in more detail in [60,67,68,76]. One of these approaches has been successfully applied in an extensive public health investigation following a “white powder” discovery in a hotel room in Las Vegas, USA, in 2008 [33]. In this context, functional methods are important to evaluate the potential danger associated with a suspect sample, thus providing decision makers with valuable information necessary to initiate appropriate countermeasures.

Generally, with respect to good analytical practices identified in the PT, it turned out that either laboratories combining different analytical approaches (*i.e.*, immunological, MS-based and functional methods) or laboratories applying highly sophisticated MS-based approaches targeting both protein identity and functional activity of ricin delivered superior results. Depending on the laboratories’ requirements appropriate methods need to be selected: exemplarily, the analysis of clinical samples requires highest sensitivity while detection of functional activity is less important. On the other hand, in the course of a forensic investigation, it might be most important to unambiguously identify the sample as ricin based on mass spectrometry including information on purity and activity of the material found. Along this line, good analytical strategies identified in this exercise used a combination of (i) highly sensitive sandwich ELISA for detection; and (ii) either LC-MS/MS or MALDI TOF MS/MS confirmation; and (iii) either an cell-based cytotoxicity assay or an MS-based adenine-release assay to highlight the functional activity of the samples. In line with Bozza *et al.* ricin-containing samples should ideally be detected and identified taking into account the biological activity of ricin’s A and B chain. Such a comprehensive analysis helps to evaluate any potential risk associated with a suspect sample and allows taking appropriate management decisions [93]. The information obtained in this PT allows future development of optimized workflows and recommended operating procedures for the analysis of ricin-containing samples—ideally this effort should be undertaken in the context of a consolidated international EQuATox network with broad expert support by different nations and standardization bodies.

With respect to quantification of ricin, several ELISA-based methods and one MS-based approach were applied. *z*-scores were used to evaluate the reported quantitative results according to [71,79]. Quantitative results reported by different ELISA-formats used in this PT showed a good mean trueness with a *z*-score mean of −0.1 for all samples. Furthermore, when evaluating results reported on highly concentrated sample S6, seven quantitative results lay within the interval −2 < *z* < +2 corresponding to satisfactory results. Therefore, the mean of participants’ quantitative results for S6 as estimated by robust statistics was defined as consensus concentration of the ricin reference material generated in [69] and used to spike PT samples in this exercise. To the best of our knowledge, this material represents the only in-depth-characterized ricin reference material available with a consensus concentration defined in an international PT. The results obtained provide a basis for further steps in quality assurance and set the basis for development of certified reference materials in the future.

The cornerstones of the next developments in the process of harmonization of analytical approaches would be to make accessible highly specific tools (mainly antibodies for extraction purposes), to thoroughly validate analytical procedures and to agree upon recommended standard operating procedures. Furthermore, further technical improvement with respect to sensitivity and specificity of some of the methods used as well as sample preparation strategies should be addressed. It can be expected that the detection limit is severely influenced or compromised in different complex matrices. Depending on the scenario and the sample under analysis, low concentrations of ricin would be expected—this is especially true for clinical samples. Finally, organizing regular proficiency tests with an increasing level of difficulty as well as ring trials on dedicated techniques will bring forward the process of harmonization and standardization and will help to keep the vigilance high.
